# ILLNESS INTRUSIVENESS AND PERCEIVED CONTROL ON QUALITY OF LIFE IN OLDER ADULTS WITH ARTHRITIS AND MULTIMORBIDITY

**DOI:** 10.1093/geroni/igae098.4215

**Published:** 2024-12-31

**Authors:** Sama Joshi, M Carrington Reid, Irina Mindlis

**Affiliations:** University of Maryland School of Medicine, Rockville, Maryland, United States; Weill Cornell Medicine, New York, New York, United States; Weill Cornell Medicine, New York, New York, United States

## Abstract

Arthritis is independently associated with diminished quality of life (QOL) among older adults; and QOL is even worse among those with arthritis and multimorbidity (MM). Illness intrusiveness—i.e., the perception that diseases and their treatments interfere with valued roles and activities—leads to poor QOL among those with chronic illnesses. Further, the perceived control individuals have over their illnesses has been proposed as a mechanism underlying the relationship between illness intrusiveness and QOL. While illness intrusiveness and perceived control are amenable to change, they are understudied among older adults with arthritis and MM. We investigated the role of perceived control as a potential mediator or moderator in the relationship between illness intrusiveness and QOL among older adults with arthritis and MM. We conducted a secondary analysis on a cross-sectional sample of older adults with MM (N=228) using PROCESS macro for SPSS. Participants were on average 72.0 years (SD=5.5), largely women (66.1%), with 2.8 chronic illnesses (SD=0.4), and high levels of pain intensity (M =7.92, SD=2.29) and illness intrusiveness (M=38.86, SD=16.55). Perceived control was a significant mediator in the relationship between illness intrusiveness and QOL, even after adjustment for pain intensity [β = -0.16, 95% CI (-0.13, -0.06)]. Perceived control did not moderate the relationship. In this sample of older adults with arthritis and MM, with high levels of illness intrusiveness and pain intensity, perceived control mediated the relationship between illness intrusiveness and QOL. Perceived control may be a target for future behavioral interventions to improve QOL in this population.

